# Evaluation of Nutritional Status and Methods to Identify Nutritional Risk in Rheumatoid Arthritis and Spondyloarthritis

**DOI:** 10.3390/nu12113571

**Published:** 2020-11-21

**Authors:** Marie Njerve Olsen, Randi J. Tangvik, Anne-Kristine Halse

**Affiliations:** 1 Mohn Nutrition Research Laboratory, Department of Clinical Medicine, Faculty of Medicine, University of Bergen, P.O. Box 7804, 5020 Bergen, Norway; randi.tangvik@uib.no; 2 Department of Rheumatology, Haukeland University Hospital, 5021 Bergen, Norway; anne.kristine.hjorteseth.halse@helse-bergen.no; 3 Department of Research and Development, Haukeland University Hospital, P.O. Box 1400, 5021 Bergen, Norway; 4 Department of Clinical Science, Faculty of Medicine, University of Bergen, P.O. Box 7804, 5020 Bergen, Norway

**Keywords:** malnutrition, nutritional risk, body composition, nutritional status

## Abstract

Patients with rheumatoid arthritis (RA) and spondyloarthritis (SpA) experience several nutritional challenges and are prone to develop malnutrition. This observational study aimed to perform a comprehensive nutritional assessment of outpatients diagnosed with RA and SpA, as well as to evaluate methods to identify nutritional risk. Nutritional status was investigated by anthropometric measures, body composition (DXA, dual energy X-ray absorptiometry), and handgrip strength (HGS). Nutritional risk was classified by Nutritional Risk Screening 2002 (NRS2002) and malnutrition was defined by the Global Leadership Initiative on Malnutrition (GLIM) criteria and fat-free mass index (FFMI; kg/m^2^, <16.7 (M), <14.6 (F)). Out of 71 included patients, 46 (66%) were abdominally obese, 28 (39%) were obese in terms of body mass index (BMI), and 33 (52%) were obese in terms of the fat mass index (FMI; kg/m^2^, ≥8.3 (M), ≥11.8 (F)). Malnutrition was identified according to FFMI in 12 (19%) patients, according to GLIM criteria in 5 (8%) patients, and on the basis of BMI (<18.5 kg/m^2^) in 1 (1%) patient. None were identified by NRS2002 to be at nutritional risk. Our study revealed high prevalence of abdominal obesity and low FFMI. Waist circumference was a good indicator of FMI. BMI, NRS2002, and HGS did not capture patients with malnutrition identified by DXA.

## 1. Introduction 

Disease-related malnutrition is a severe life-threatening condition receiving considerable and increasing attention in healthcare. Malnutrition has been shown to reduce patients’ resistance to infections and quality of life, and increase the risk of morbidity, hospitalization, and even mortality [1,2]. National and international nutrition guidelines, as well as patient safety campaigns, emphasize procedures to improve nutritional status in hospitalized patients, starting with nutritional risk screening [3]. In cases of nutritional risk, nutritional assessment is recommended, and, in most cases, an individualized nutritional plan should be made. As many as one in three hospitalized patients are at nutritional risk [2,4]. An individualized nutritional plan has been shown to improve clinical outcome and survival for these at-risk patients [5,6].

Patients with rheumatoid arthritis (RA) and spondyloarthritis (SpA) are prone to develop malnutrition as their medication often affects appetite, nutrient absorption, and digestion [7,8,9,10,11]. Due to joint pain and fatigue, patients can experience challenges in food shopping and preparation [8,12]. In addition, the increased level of inflammation may increase nutritional needs [13]. Nutritional status is commonly assessed by body mass index (BMI; kg/m^2^) in a clinical setting [14]. However, reduced fat-free mass (FFM) and increased fat mass (FM) have been reported in rheumatic patients, due to cytokine-driven hyper-metabolism and decreased activity levels [15]. Rheumatoid cachexia (RC) is a term used to describe this phenomenon [16]. An increase in FM often masks a reduction in FFM, which results in no change in BMI. Measurement of body composition (fat-free mass index (FFMI; kg/m^2^) and fat mass index (FMI; kg/m^2^)) provides more precise information, and dual energy X-ray absorptiometry (DXA) is considered the gold standard for this purpose [14]. FFMI and FMI are conceptually similar to BMI (BMI = FFMI + FMI). There is no clear consensus on the cut-off levels used to define RC, but FFMI <10th percentile and FMI >25th percentile have been proposed [17].

Although new drugs have revolutionized treatment, some patients still suffer from pain, stiffness, or fatigue, or experience side effects of their drugs. Patients with rheumatic diseases are therefore likely to experiment with “dietary therapies” as a complementary treatment. However, the scientific evidence regarding the effect of specific diets on RA and SpA is limited [18,19]. It is a cause for concern that dietary therapies, leading to restrictions that are not based on scientific evidence, can lead to reduced intake of food or nutrients [20].

As treatment of inflammatory rheumatic diseases in hospitals increasingly takes place in outpatient clinics, it is necessary to validate the nutritional screening tools and guidelines for this purpose. To our knowledge, no such guidelines exist for patients with RA or SpA [21]. Further, it is unclear to what extent Nutritional Risk Screening 2002 (NRS2002) identifies nutritional risk in this patient group [22,23]. It is worrying that little attention is given to these patients’ dietary intake and nutritional status [24,25]. The Global Leadership Initiative on Malnutrition (GLIM) has developed a two-step approach for diagnosing malnutrition: (i) a nutritional risk screening using a validated screening tool (e.g., NRS2002) and (ii) a diagnostic assessment that considers both phenotypic and etiological aspects of malnutrition [26]. It is unclear whether these criteria are appropriate to identify malnutrition in patients with RA and SpA. The majority of previous studies addressing nutrition and rheumatic disease have applied to patients with RA, while studies on SpA are rare [19].

Therefore, this study aimed to evaluate nutritional status and dietary habits in outpatients with RA and SpA in order to describe nutritional challenges for these patients. We also wanted to investigate whether nutritional risk screening and the GLIM criteria are valid to evaluate nutritional status of outpatients with RA and SpA by comparing these with more comprehensive methods. 

## 2. Materials and Methods 

### 2.1. Study Design and Setting

This was an observational study conducted in the Rheumatology Department of Haukeland University Hospital (HUH) from August 2018 to December 2019. HUH is the second largest hospital in Norway with about 1000 beds. All data were collected by master students in clinical nutrition (Marie Njerve Olsen and Mathilde Skogseide), unless otherwise stated.

### 2.2. Study Population and Recruitment 

Outpatients taking part in a rehabilitation program and outpatients receiving infusions with biological disease-modifying anti-rheumatic drugs (bDMARDs) were eligible for inclusion. Patients in the rehabilitation program were invited to participate upon arrival, while those receiving bDMARDs were invited prior to hospitalization. 

Inclusion criteria were adult patients diagnosed with one of the following diagnoses, according to the 10th version of the International Classification of Diseases: rheumatoid arthritis (RA) (M05.8, M05.9, M06.0), polyarthritis (M13.0), psoriatic arthritis and enteropathic arthritis (M07.0, M07.3, M07.4, M07.5), ankylosing spondylitis (M45), sacroiliitis (M46.1), and unspecified spondyloarthritis (M46.8, M46.9). For practical reasons, patients with RA and polyarthritis were categorized as RA. Patients with psoriatic arthritis, ankylosing spondylitis, sacroiliitis, and unspecified spondyloarthritis were categorized as SpA. We excluded patients with severe cardiovascular diseases, such as heart failure (NYHA class II-III), neurological diseases, severe mental illnesses, pregnant and lactating women, and non-Norwegian-speaking patients. 

### 2.3. Data Collected

#### 2.3.1. Demographics

Information about socioeconomic status, level of physical activity, and the need for help with housekeeping were collected through a questionnaire. Diagnosis, disease duration, and use of medication were recorded in the data program “GoTreatITRheuma" by the physician. The medications were categorized into the following four groups: disease-modifying anti-rheumatic drugs (DMARDs), non-steroidal anti-inflammatory drugs (NSAIDs), glucocorticoids (GC), or none. 

#### 2.3.2. Nutritional Assessment

The anthropometric measurements height (m), weight (kg), and waist circumference (WC; cm) were obtained according to guidelines [27,28,29]. Height was measured by a wall-mounted stadiometer, without shoes with heels placed against the wall and the head positioned in the Frankfort plane [27]. Weight was measured in light clothing with TANITA MC-180MA, Tanita Corporation, Tokyo, Japan. One kilogram, was subtracted from the weight for the clothes. WC was measured by a non-stretchable measuring tape at the mid-point between the lowest palpable rib and the iliac crest. 

Body composition included FM (kg) and FFM (kg), measured with dual-energy X-ray absorptiometry (DXA) in 63 patients, standardized by qualified personnel (Lunar iDXA Scanner, GE Medical Systems Lunar, Madison WI, USA). In addition, body mass index (BMI; kg/m^2^), fat-free mass index (FFMI; FFM in kg/m^2^), and fat mass index (FMI; FM in kg/m^2^) were calculated. Underweight, overweight, and obesity were classified according to the BMI categories of the World Health Organization [30]. Levels of FMI and FFMI (DXA) were compared with levels in a healthy European population [31]. Levels were categorized as low, normal, high, or very high, depending on the matching level reported in the BMI categories as previously described by Kyle et al. [31].

Blood samples for analysis of nutritional and inflammatory biomarkers were collected by a study nurse, analyzed at the laboratory of HUH, and compared with levels in healthy age- and sex-matched adults [32]. 

Handgrip strength (HGS) was measured by a dynamometer (maximum weight capacity of 80 kg) (KERN MAP 80K1, Kern and Sohn, Balingen, Germany), according to guidelines [33]. The mean value of 3 results was compared with levels in healthy age- and sex-matched adults. A level below the 5th percentile was considered to be low HGS [33].

#### 2.3.3. Malnutrition and Nutritional Risk Screening

Malnutrition was defined by muscle mass (FFMI <16.7 (M) and <14.6 (F), measured by DXA) and the GLIM criteria [26,31]. In order to receive a malnutrition diagnosis according to the GLIM criteria, patients must fulfil 1 phenotypic and 1 etiological criterion. The phenotypic criteria are involuntary weight loss, BMI <20 or <22 if ≥70 years, and reduced muscle mass, FFMI <15 for males or <17 for females. The etiological criteria are reduced food intake or assimilation, and inflammation [26]. 

Nutritional risk was defined as a score of 3 or more points on NRS 2002, while rheumatoid cachexia (RC) was defined as FFMI below the 10th percentile combined with FMI above the 25th percentile in a healthy reference population [26,34].

#### 2.3.4. Dietary Habits

Information about dietary knowledge, dietary restrictions, and use of supplements was collected through a validated questionnaire [35]. In addition, questions about dietary habits were included.

#### 2.3.5. Ethics

The study was approved by the Norwegian “Regional Committees for Medical and Health Research Ethics”, 2018/1063 (07/06/18), and conducted in accordance with the Declaration of Helsinki. Written informed consent was obtained from the participants. The participants could withdraw at any time without consequences for their further treatment. 

### 2.4. Statistical Analysis

Statistical analysis was performed in SPSS version 25 (SPSS Inc. Chicago, IL, USA). Statistical power was not calculated due to the study design. The normal distribution of the continuous variables was tested by the Shapiro–Wilk test. Continuous variables are reported as mean with standard deviation, minimum and maximum values. Categorical variables are reported as frequencies and percentages.

A chi-squared test with Yates’ continuity correction was performed to compare the groups. 

We calculated sensitivity, specificity, positive predictive value, and negative predictive value for NRS2002, categories of BMI, WC, and HGS in order to identify malnutrition, defined as low FFMI (<16.7 (M) or <14.6 (F)), and obesity, defined as very high FMI (≥8.3 (M) or ≥11.8 (F)), measured by DXA [31]. *p*-values < 0.05 were considered significant. 

## 3. Results

### 3.1. Description of the Study Population

A total of 71 outpatients fulfilled the inclusion criteria and participated in the study: 53 (75%) females, mean age 52 ± 12 years and mean BMI 28.0 ± 5.5 kg/m^2^. The mean disease duration was 14 ± 12 years. Table 1 shows the characteristics of the study population.

Eleven (15%) patients had a gastric condition: celiac disease (*n* = 1), previous bariatric surgery (*n* = 3), or inflammatory bowel disease (*n* = 7). A total of 34 (48%) patients were diagnosed with RA, and 37 (52%) patients had SpA. Fifty-seven (80%) patients used one or more DMARDs, and the rest used either NSAIDs or GC.

### 3.2. Nutritional Assessment

Table 2 shows data on nutritional status.

Abdominal obesity was present in 46 (66%) patients, and 46 (65%) were overweight or obese, on the basis of BMI.

A very high FMI by DXA was seen in 33 (52%) of the 63 patients, measured by DXA (Table 3). High or very high FMI was found in 12 (52%) of the 23 patients with normal BMI (Table 3). The mean value of FMI was higher than the reference values for males and females (Table 2).

Vitamin D deficiency (<50 nmol/L) was observed in 13 (19%) patients, and 12 (17%) patients had a low level of hemoglobin (<13.4 g/dL (M), <11.7 g/dL (F)). 

Compared with the reference levels of healthy adults, 19 (30%) patients had lower HGS in the dominant hand, of whom 13 patients (42%) had RA and 6 (18%) had SpA. Low HGS in the non-dominant hand was observed in eight (13%) patients. 

### 3.3. Malnutrition and Nutritional Risk Screening

Low FFMI, by DXA, was observed in 12 (19%) patients, of which 7 had RA and 5 SpA. None of the malnourished participants were identified to be at nutritional risk according to NRS 2002, and five (8%) patients were moderately malnourished according to GLIM criteria. One (2%) patient was underweight in terms of BMI. Furthermore, nine (14%) patients fulfilled the criteria for RC (FFMI <10th percentile and FMI >25th percentile), of which four were also malnourished according to GLIM criteria. 

Levels of FMI and FFMI varied greatly within the BMI categories, and low FFMI was found in 11 of the 23 (36%) patients with a normal BMI (Table 3).

No significant differences between RA and SpA in terms of low FFMI, low HGS, obesity, or abdominal obesity were observed. 

### 3.4. Evaluation of Methods to Detect Malnutrition and Obesity

Compared with DXA, low HGS had higher sensitivity in detecting malnutrition (63%) than low BMI, defined by GLIM criteria (42%), BMI < 18.5 (8%), and NRS2002 (0%) (Table 4).

Abdominal obesity had the highest sensitivity in detecting a very high level of FMI, but had lower specificity than the BMI categories ≥28 and ≥30 (67%) (Table 5).

### 3.5. Dietary Habits

The questionnaire revealed that dietary supplements were used by 50 (70%) patients; fish oil or omega-3 supplements were most common, used by 32 (45%). Dietary restrictions were reported by 29 (41%) patients. Most frequently excluded or reduced were pork (10 patients, 34%), wheat (10 patients, 34%), and dairy products (9 patients, 31%). In addition, eight (28%) patients reported avoiding sugar.

Lack of knowledge of nutrition and diet was reported by 24 (34%) patients, while 32 (46%) patients reported a dietary change to reduce their disease activity. Out of 37 patients with SpA, 20 (54%) reported a dietary change.

## 4. Discussion

This study provides a comprehensive nutritional assessment of outpatients diagnosed with RA and SpA. Most of the patients were overweight or obese with high occurrence of abdominal fat mass. Importantly, one in five had a low FFMI. Even patients with normal BMI had a low FFMI, classified as malnourished. Notably, none of the patients were identified to be at nutritional risk on the basis of NRS2002. Low HGS showed highest sensitivity in detecting malnutrition, but also the lowest specificity. Patients reported poor nutritional knowledge.

In this study, the prevalence of abdominal obesity (WC) and obesity (BMI) was higher than in the general Norwegian population (66% vs. 45% and 39% vs. 20–25%, respectively) [36,37]. We found a higher percentage of obesity (BMI) than in other studies of RA and SpA patients (39% vs. 13–22%) [22,38]. The prevalence of obesity tends to increase during the progression of the disease [39]. Nikiphorou et al. reported an annual increase in BMI of 0.27 units in newly diagnosed RA subjects in a five-year follow-up study [25]. Investigations of body composition with DXA revealed that the WHO categories of BMI were not a sufficient indicator of overweight, as almost half of the patients with normal BMI had high FMI. New BMI cut-offs have been proposed to define overweight (>23 kg/m^2^) and obesity (>28 kg/m^2^) in patients with inflammatory rheumatic diseases (IRD) [40]. Using the suggested cut-off values, half of our patients would be categorized as obese. However, WC predicted high fat mass even more precisely than BMI. WC is easy to perform in a clinical setting and is also an important risk factor for cardiovascular disease. Our results concur with those of previous studies reporting altered body composition in patients with IRD [41]. Both the disease and medication might affect body composition. In particular, glucocorticosteroids increase appetite and influence body composition by increasing WC and reducing muscle mass [42]. Since obesity is associated with higher disease activity, lower probability of remission, and poorer treatment response to medication, management of obesity is of utmost importance [25,43]. 

Only one patient in the entire study population had low BMI, but interestingly, one-fifth of the patients had a low FFMI and one in seven had RC. In contrast to other studies, none of our patients had experienced involuntary weight loss within the last 6 months [44]. Thus, screening tools including BMI as one of the criteria for nutritional risk are not reliable.

We found a lower prevalence of RC than previous studies—14% vs. 20–38% [44,45]. A possible explanation might be the selection of patients, due to close follow-up at the outpatient clinic, and they may therefore have had lower disease activity than reported in older studies [44].

The key finding that half of our patients with normal BMI had low FFMI has previously been reported in a study by Konijn et al., where 38% of patients with normal BMI had low FFMI, measured by bioelectrical impedance analysis [41]. Neither screening tools nor BMI are suitable for detecting malnutrition and RC in patients with RA [17,22]. Thus, BMI < 18.5 may be too low to identify nutritional risk in patients with RA and SpA. Our results indicate that the cut-off values for low BMI (<20/22), based on the GLIM criteria, are more sensitive in identifying nutritional risk. 

Low HGS was sensitive in detecting low FFMI, although PPV was low. HGS has been found to correlate with functional status and muscle mass in hospitalized patients [46]. On the other hand, HGS can probably not be used as a functional marker of nutritional status in this patient group due to joint pain and reduced hand function [47]. This is confirmed by the low PPV of HGS, meaning that a low HGS does not indicate a high probability of malnourishment. 

Dietary supplements were more frequently used in our patients than in the general Norwegian population (70% vs. 53%), and also more often than in IRD in previous studies [48,49]. Female gender and high education are associated with the use of dietary supplements and might explain the frequent use of supplements in our study population [50]. 

Dietary restrictions might lead to insufficient intake of important nutrients [20]. Half of the patients reported having altered their diet to reduce disease symptoms, mostly limiting foods high in calcium and proteins. Proteins are important to preserve muscle mass, and calcium to prevent osteoporosis, both of relevance for patients with IRD. However, one in three patients reported a lack of relevant nutritional knowledge. Thus, individual dietary guidance might help patients to ensure the quality of their diet, achieve weight control, and prevent comorbidities.

### 4.1. Strengths and Limitations

This was a cross-sectional observational study, and our results have certain limitations. Bowel inflammation is more common in patients diagnosed with SpA, and might have affected the absorption of nutrients [51,52]. We did not exclude any patients with conditions affecting nutritional status. Patients with an interest in nutrition and health might have been over-represented among those who consented to study participation. A clear weakness is that we did not focus on previous use of medication, which can affect nutritional status. It is also a limitation that the questionnaire used in this study was not tested in a pilot. 

A major strength is that DXA was used to measure FM and FFM [17,53]. The comprehensive assessment of nutritional status strengthens internal validity, as several parameters were used to judge nutritional status. The majority of questions were based on previously validated questionnaires.

### 4.2. Clinical Implications

In this study, the nutritional situation of patients with RA and SpA was complex. Both malnutrition and obesity were present, and low nutritional knowledge was reported. Hence, these patients may benefit from dietary counselling. Early nutritional risk screening and assessment of nutritional status are important. However, existing screening tools are not appropriate for these patients with altered body composition.

The GLIM involves a two-step approach for diagnosing malnutrition [26]. It is a concern that patients identified as malnourished in step 2 were not identified in step 1, as 20% were malnourished on the basis of low muscle mass, and none according to NRS2002. We therefore suggest excluding the first screening step in the GLIM criteria for this patient group. 

### 4.3. Further Research 

Due to the complex nutritional status of these patients, they may be expected to benefit from optimization of their diet. No specific dietary recommendations for patients with IRD exist, and existing screening tools for identifying nutritional risk are not appropriate. Many patients may receive information from unvalidated sources. Further studies should address these issues. The effect of diet on disease activity remains unclear, and we are currently conducting an intervention study to examine this.

## 5. Conclusions

In summary, pronounced nutritional characteristics of patients with RA and SpA were abdominal obesity and low muscle mass. We suggest that WC is a reliable indicator of FMI. Neither BMI, NRS2002, nor HGS captured patients with malnutrition, identified by DXA. Studies to investigate other screening tools to identify risk of malnutrition in this patient population are needed. 

## Figures and Tables

**Table 1:** General and socioeconomic characteristics of the study population; *N* = 70.

	*n* (%)
*Education*	
Primary or high school, or certificate of apprenticeship	23 (33)
College/university	47 (67)
*Work situation*	
Paid work	45 (64)
*Tobacco use*	
Current smoker	12 (17)
Former smoker	29 (41)
Uses snuff	6 (9)
*Physical activity level*	
≤3 times/week	47 (67)
Almost every day	23 (33)
*Needs help with housekeeping*	
Sometimes	28 (39) *^a^*
Not at all	43 (61) *^a^*

*^a^n* = 71.

**Table 2:** Anthropometric measurements, body composition, biochemical assessments, and handgrip strength; *N* = 71.

Variable		*N*	Mean ± SD	Min–Max	Reference Value	Below Reference *n* (%)	Above Reference *n* (%)
Anthropometry							
BMI (kg/m^2^)	All	71	28.0 ± 5.5	18.2–39.9	18.5–24.9	1 (1)	46 (65)
WC (cm)	M	18	103.8 ± 14.0	82.0–134.0	<94	3 (17)	15 (83)
	F	52	98.6 ± 15.5	67.5–130.5	<80	5 (10)	47 (90)
Body composition							
FFMI (kg/m^2^)	M	17	19.8 ± 1.9	14.8–22.7	16.7–19.8	1 (6)	8 (47)
	F	46	16.2 ± 1.9	12.2–20.7	14.6–16.8	11 (24)	15 (33)
FMI (kg/m^2^)	M	17	9.0 ± 3.3	4.0–15.0	1.8–5.2	0	15 (88)
	F	46	11.8 ± 4.4	4.5–21.7	3.9–8.2	0	37 (80)
Biochemical parameters							
Hemoglobin (g/dL)	M	18	14.4 ± 1.1	13.0–16.2	13.4–17.0	5 (28)	0
	F	53	13.0 ± 0.9	10.9–15.3	11.7–15.3	7 (13)	0
Ferritin (µg/L)	M	18	140.4 ± 83.2	43.0–321.0	34–300	0	1 (6)
	F	52	102.4 ± 56.1	8.0–244.0	18–240	2 (4)	1 (2)
Homocysteine (µmol/L)	All	70	10.9 ± 3.0	5.2–19.7	<15	-	8 (11)
Methylmalonic acid (µmol/L)	All	70	0.16 ± 0.1	0.1–0.4	<0.26	-	6 (9)
Vitamin D (nmol/L)	All	70	66.8 ± 19.6	19.0–111.0	50–113	13 (19)	0
Zinc (µmol/L)	All	70	12.2 ± 1.8	8.5–16.7	10.1–16.6	9 (13)	1 (1)
Erythrocyte sedimentation rate (mm/t)	M	18	11.3 ± 11.2	1.0–44.0	1–20 *^a^*	-	3 (17)
	F	51	13.5 ± 11.6	1.0–43.0	1–30 *^a^*	-	5 (10)
Handgrip strength							
Dominant hand	M	16	35.8 ± 10.6	18.5–59.0	>5pct *^b^*	2 (13)	14 (87)
	F	48	17.6 ± 7.0	4.9–40.1	>5pct *^b^*	17 (35)	31 (65)
Non-dominant hand	M	16	32.4 ± 10.1	19.2–53.3	>5pct *^b^*	3 (19)	13 (81)
	F	44	17.3 ± 6.4	6.8–41.5	>5pct *^b^*	5 (11)	39 (89)

*^a^* Age-specific references exist. *^b^* Handgrip strength below 5th percentile in healthy age- and sex-matched adults was considered to be below reference [33].

**Table 3:** Distribution of low, normal, high, and very high levels of fat mass index and fat-free mass index within the BMI categories; *N* = 63.

Variable	Underweight	Normal Weight	Overweight	Obese	Total
	BMI < 18.5 *^a^*	BMI 18.5–24.9 *^a^*	BMI 25–29.9 *^a^*	BMI ≥ 30 *^a^*	
	*n* (%)	*n* (%)	*n* (%)	*n* (%)	*n* (%)
Low FMI *^a^*	-	-	-	-	-
Normal FMI *^a^*	1 (2)	11 (17)	-	-	12 (19)
High FMI *^a^*	-	11 (17)	7 (11)	-	18 (29)
Very high FMI *^a^*	-	1 (2)	7 (11)	25 (40)	33 (52)
Low FFMI *^a^*	1 (2)	11 (17)	-	-	12 (19)
Normal FFMI *^a^*	-	10 (16)	12 (19)	7 (11)	29 (46)
High FFMI *^a^*	-	2 (3)	2 (3)	8 (13)	12 (19)
Very high FFMI *^a^*	-	-	-	10 (16)	10 (16)
Total *n* (%)	1 (2)	23 (36)	14 (22)	25 (40)	63 (100)

*^a^* kg/m^2^. Body composition measured by dual-energy X-ray absorptiometry. Low, normal, high, and very high fat mass index (FMI; kg/m^2^), and fat-free mass index (FFMI; kg/m^2^) defined as levels reported in a healthy European population with body mass index (BMI) of <18.5 kg/m^2^, 18.5–24.9 kg/m^2^, 25–29.9 kg/m^2^, and ≥30 kg/m^2^, respectively [31]. Low FMI: <1.8 (M), <3.9 (F). Normal FMI: 1.8–5.2 (M), 3.9–8.2 (F). High FMI: 5.2–8.3 (M), 8.2–11.8 (F). Very high FMI: ≥8.3 (M), ≥11.8 (F). Low FFMI: <16.7 (M), <14.6 (F). Normal FFMI: 16.7–19.8 (M), 14.6–16.8 (F). High FFMI: 19.8–21.7 (M), 16.8–18.2 (F). Very high FFMI ≥21.7 (M), ≥18.2 (F).

**Table 4:** Sensitivity, specificity, positive predictive value, and negative predictive value of malnutrition identified by BMI, Nutritional Risk Screening 2002 (NRS2002), and handgrip strength (HGS); *N* = 63.

	Malnourished *^a^*	Well-Nourished	Sensitivity	Specificity	PPV	NPV
	(*n*)	(*n*)	(%)	(%)	(%)	(%)
BMI <18.5 *^b^*			8	100	100	82
Yes (*n*)	1	0				
No (*n*)	11	51				
BMI <20, or <22 if ≥70 y *^c^*			42	100	100	88
Yes (*n*)	5	0				
No (*n*)	7	51				
At nutritional risk *^d^*			0	100	0	81
Yes (*n*)	0	0				
No (*n*)	12	51				
Low HGS			63	73	28	92
Yes (*n*)	5	13				
No (*n*)	3	36				

PPV: positive predictive value. NPV: negative predictive value. *^a^* Malnourished patients defined as fat-free mass index (FFMI; kg/m^2^) < 16.7 (M), <14.6 (F), measured by DXA. *^b^* kg/m^2^. *^c^* Defined as low BMI by the Global Leadership Initiative on Malnutrition. *^d^* NRS2002: Nutritional Risk Screening 2002 ≥3. Low handgrip strength (HGS) <5 pct in age- and sex-matched healthy adults [33].

**Table 5:** Sensitivity, specificity, positive predictive value, and negative predictive value of obesity identified by BMI and waist circumference (WC); *N* = 63.

	Obese *^a^*	Not Obese	Sensitivity	Specificity	PPV	NPV
	(*n*)	(*n*)	(%)	(%)	(%)	(%)
BMI ≥ 30 *^b^*			76	100	100	79
Yes (*n*)	25	0				
No (*n*)	8	30				
BMI ≥ 28 *^b^*			91	97	97	90
Yes (*n*)	30	1				
No (*n*)	3	29				
Abdominal obesity *^c^*			97	67	76	95
Yes (*n*)	31	10				
No (*n*)	1	20				

PPV: positive predictive value. NPV: negative predictive value. *^a^* Obese patients defined as very high fat mass index (FMI; kg/m^2^): ≥8.3 (M), ≥11.8 (F), measured by DXA. *^b^* kg/m^2^. *^c^* Abdominal obesity: waist circumference ≥102 cm (M), ≥88 cm (F).
